# Oxidative Stress in Liver Diseases: Pathogenesis, Prevention, and Therapeutics

**DOI:** 10.1155/2017/8341286

**Published:** 2017-04-25

**Authors:** Ravirajsinh N. Jadeja, Ranjitsinh V. Devkar, Srinivas Nammi

**Affiliations:** ^1^Division of Gastroenterology/Hepatology, Department of Medicine, Medical College of Georgia, Augusta University, Augusta, GA 30912, USA; ^2^Division of Phytotherapeutics and Metabolic Endocrinology, Department of Zoology, Faculty of Science, The Maharaja Sayajirao University of Baroda, Vadodara, Gujarat 390002, India; ^3^School of Science and Health, Western Sydney University, Penrith, NSW 2751, Australia; ^4^National Institute of Complementary Medicine (NICM), Western Sydney University, Penrith, NSW 2751, Australia

Reactive oxygen species- (ROS-) induced oxidative stress has been implicated in various forms of diseases. Excessive ROS generation depletes the endogenous antioxidants that subsequently fail to counteract all the ROS leading to cellular injury. Alcohol consumption, high-calorie diet, drug overdose, environmental pollutants, heavy metals, and so forth have been implicated towards manifestation of liver injury via generation of ROS. Hepatocyte mitochondria and endoplasmic reticulum are the major site for ROS generation in various forms of liver diseases. Hence, antioxidants are frequently used to treat oxidative liver injury. Although preclinical results are promising for antioxidant therapy in treating liver diseases, evidences obtained from clinical studies are unclear. Further, detailed investigation on the mechanism of ROS-mediated hepatocyte injury and protective role of antioxidants could provide insight into pathogenesis of hepatocyte injury and open new avenues for diagnosis and development of biomarkers and pharmacological therapies. This special issue is a complication of five preclinical research articles and a review accepted from many submissions following a peer review process.

Cholesterol has emerged as a critical player in liver injury. N. Nuño-Lámbarri et al. evaluated the impact of liver cholesterol overload on the progression of the obstructive cholestasis in mice induced by bile duct ligation (BDL) surgery. The results showed that high cholesterol- (HC-) fed mice exhibited augmented oxidative stress and apoptosis along with reduced proliferation of hepatocytes. Further, mortality following BDL was higher in HC-fed mice compared to sham-operated mice. Authors concluded that hepatic cholesterol accumulation impairs hepatocyte regeneration during obstructive cholestasis and aggravates the disease with early fatal consequences. In a study by F. Li et al., aged male *Sprague Dawley* rats were exposed to treadmill training with medium intensity and preatomic analysis of liver extract was carried out. Results obtained from two-dimensional gel electrophoresis followed by MS/MS identification indicated changes in several antioxidants. The expression of several proteins involved in key liver metabolic pathways including mitochondrial sulfur, glycolysis, methionine, and protein metabolism was altered compared to that of control rats. These findings indicate that exercise may be beneficial to aged rats through modulation of hepatic protein expression profiles. A beneficial effect of n-3 polyunsaturated fatty acids (PUFAs) in a model of liver disease was evaluated by R. Feng et al. They used fat-1 transgenic mice that synthesize endogenous n-3 from n-6 PUFA. Fat-1 and their WT littermates were exposed to a modified AIN93 diet containing 10% corn oil followed by intraperitoneal injection of a single dose of carbon tetrachloride. Their results showed that severe liver injury in WT mice was remarkably ameliorated in fat-1 mice. They attributed these effects to the activation of Nrf2/keap1 pathway. These findings indicate that n-3 PUFA has potent protective effects against acute liver injury and can be explored for other forms of liver diseases.

The fungus *Antrodia cinnamomea* (AC) is traditionally been used in China for various health benefits. A study by Y. Liu et al. not only systematically analyzed the components of fermented AC but also demonstrated its protective effect against alcohol-induced hepatocyte injury. Treatment of acute ethanol-intoxicated mice with AC extract significantly improved levels of AST and ALT activity, oxidation-related enzymes, inflammatory cytokines, and caspases. They also concluded that these effects might be via Akt/NF-κB signaling pathway. Garlic is well known for its beneficial effects against many diseases, including liver ailments. A study by F. Zhang et al. evaluated the underlying mechanism for reduced live fibrosis by diallyl trisulfide (DATS), the primary organosulfur compound in garlic. The primary rat hepatic stellate cells (HSCs) were treated with hydrogen peroxide (H_2_O_2_), and profibrogenic and oxidative stress parameters were evaluated. The results showed that DATS reduced fibrotic marker expression in HSCs. Further, DATS arrested cell cycle at G2/M checkpoint associated with downregulation of cyclin B1 and cyclin-dependent kinase 1, induced caspase-dependent apoptosis, and reduced migration in HSCs. Moreover, intracellular levels of reactive oxygen species and lipid peroxide were decreased by DATS. Authors concluded that DATS mediated its effects via H_2_S generation. In hepatocytes, oxidative stress frequently triggers antioxidant response by activating nuclear erythroid 2-related factor 2 (Nrf2), a transcription factor, which upregulates various cytoprotective genes. Many studies have highlighted Nrf2 as a potential therapeutic target to treat liver diseases. A review article by R. N. Jadeja et al. comprehensively appraises various phytochemicals that have been assessed for their potential to halt acute and chronic liver injury by enhancing the activation of Nrf2 and have the potential for use in humans.

